# 594. Micafungin Prophylaxis in Acute Myeloid Leukemia Adult Patients Undergoing Induction Chemotherapy

**DOI:** 10.1093/ofid/ofab466.792

**Published:** 2021-12-04

**Authors:** Justine Abella Ross, Jonathan Yong, Jason Chen, Deron Johnson, Doreen Pon, Sanjeet Dadwal

**Affiliations:** City of Hope National Medical Center, Duarte, CA

## Abstract

**Background:**

Patients (pts) with newly diagnosed acute myeloid leukemia (AML) undergoing induction chemotherapy are at increased risk for invasive fungal infections (IFI). Guidelines recommend posaconazole prophylaxis (ppx), but use is precluded by interactions and adverse effects. Micafungin (MCF) is an alternative, but data is limited by small prospective and retrospective studies. Primary objective: describe incidence of probable/proven IFI until neutrophil recovery (ANC ≥ 500 cells/µL) or 28 days after induction start date, whichever occurred first, in pts receiving MCF ppx. Secondary objective: describe incidence of clinical failure to MCF prophylaxis.

**Methods:**

Retrospective review (January 2017 to January 2020) of newly diagnosed AML adult pts undergoing 7 + 3 using idarubicin (7 + 3-ida), 7 + 3 using daunorubicin (7 + 3-dau), venetoclax/decitabine (VEN/DEC), or venetoclax/azacitadine (VEN/AZA) receiving MCF ppx for at least 7 days included. Diagnosis of IFI < 30 days prior to induction, liver function tests (LFT) 5x ULN at start of induction, or evidence of refractory disease after induction excluded. Probable/proven IFI defined by EORTC criteria. Clinical failure: changing to a different antifungal class for any reason until ANC recovery or 28 days after induction start date.

**Results:**

Ninety-five pts included. Baseline characteristics: mean (±SD) age 57.8 (±13.0) years; 53.6% males. 62% (59/95) 7 + 3-ida, 13.7% (13/95) 7 + 3-dau, 15.8% (15/95) VEN/DEC, 8.4% (8/95) VEN/AZA. Mean (±SD): 32.5% (±26) blasts, WBC 13.2 (±23.8), ANC 2.4 (±4.6), ALC 1.9 (±1.6), platelets 92.6 (±123.2). Incidence of probable IFI 2/95 (2.1%). No proven IFI cases identified. Clinical failure occurred in 37/95 (39%): 8 persistent febrile neutropenia, 29 due to suspected IFI. No MCF discontinuation due to adverse events.

**Conclusion:**

Our findings suggest that prophylactic MCF is safe and effective in pts with newly diagnosed AML undergoing induction chemotherapy. Outcomes were similar to those of prophylactic posaconazole studies, indicating MCF may be considered as an alternative when interactions and adverse effects preclude use of posaconazole. Our study was limited by small numbers, retrospective, single-center design. Future opportunities include prospective trials of prophylactic MCF in this setting.

**Disclosures:**

**All Authors**: No reported disclosures

